# Supplementary material for: Quantitative Insights into Pressure-Dependent Mass Transport and Reaction Kinetics in Electrochemical CO2 Reduction
Source: JACS Au. 2026 May 20;6(6):3515–26. doi: 10.1021/jacsau.6c00536 (PMC13291964; doi:10.1021/jacsau.6c00536)
Supplement: Supplementary file 1 [file au6c00536_si_001.pdf]

Supporting Information

Quantitative insights into pressure-dependent mass transport and reaction kinetics in electrochemical CO_2 reduction

Mengtian Jin,[1,2#] Ouwen Peng,[3#] Yanrong Xue,[1,2] Jiahao Liu,[1,2] Lian Tang,[1,2] Xu Lu[1,2*]

1 Clean Energy Research Platform (CERP), Division of Physical Science and Engineering (PSE), King Abdullah University of Science and Technology (KAUST), Thuwal 23955-6900, Kingdom of Saudi Arabia
2 Center for Renewable Energy & Storage Technologies (CREST), Division of Physical Science and Engineering (PSE), King Abdullah University of Science and Technology (KAUST), Thuwal 23955-6900, Kingdom of Saudi Arabia
3 Illinois Advanced Research Center at Singapore (Illinois ARCS),1 CREATE Way, Singapore 138602, Singapore
# These authors contributed equally.
* Corresponding author. Email: Xu Lu (xu.lu@kaust.edu.sa).

Materials and methods

Materials

Polyvinylpyrrolidone (PVP, MW =50000), glucose, silver nitrate ($AgNO_3$, 99.0%), sodium hydroxide (NaOH, 99.99%), potassium bicarbonate ($KHCO_3$, 99.99%), and potassium sulfate (K_2SO_4, 99.0%) were purchased from Sigma Aldrich. All the chemicals were used directly without any additional purification.

Electrode Preparation

For catalyst preparation, silver nanoparticles (Ag NPs) were synthesized using a chemical reduction method.[1] First, 1 g PVP was dissolved in 20 mL of deionized water. Then, 10 mL of 0.1 M aqueous NaOH solution and 10 mL of 0.2 M aqueous glucose solution were consecutively added to the PVP solution. Once fully dissolved, 20 mL of 0.02 M aqueous $AgNO_3$ solution was dropwise added to the mixed solution. The reaction mixture was then stirred for an additional 10 min before being heated to 60 °C and maintained at this temperature for 60 min. Afterwards, the solution was cooled to room temperature. The suspension was centrifuged at 8000 rpm and washed three times with deionized water. The purified Ag NPs were then stored in ethanol for subsequent use.
The electrode preparation as follows: 1 ml of ethanol solution containing 6 mg of obtained Ag NPs and 40 μL of Nafion solution (5 wt%, Sigma-Aldrich) was sonicated for 60 min. Next, the catalyst slurry was sprayed onto the carbon paper (Toray 120, Fuel Cell Store, 0.30 mm thick) by the airbrush at 50 °C. The catalyst mass loading is 3 mg cm^{-2}.

Structural characterization

Scanning electron microscopy (SEM) images were acquired using a Quattro microscope operated at 20 kV. Transmission electron microscopy (TEM) images were obtained with an FEI Tecnai G2 Spirit Twin microscope, operated at 120 kV. X-ray photoelectron spectroscopy (XPS) analysis was performed using a Kratos Analytical AMICUS/ESCA 3400 instrument, equipped with an Mg-anode Kα excitation X-ray source, operating at 10 kV, 10 mA, and a pressure of 2×10^{-6} Pa. X-ray powder diffraction (XRD) patterns were collected using a Bruker D8 Advance diffractometer with Cu Kα radiation.

Electrochemical measurements

High-pressure electrochemical measurements were conducted using a previously reported high-pressure H-cell configuration (Tianjin Jike Xingze Technology Co., Ltd.).[2] The cell is comprised of two chambers, each filled with 50 mL of 0.5 M $KHCO_3$ electrolyte and is separated by a proton exchange membrane (Nafion-117, Fuel Cell Store). The working electrode (1 × 1 cm^2) and the Ag/AgCl reference electrode were placed in the cathode chamber, and the counter electrode (Pt foil, 1 × 1 cm^2) was placed in the anode chamber. Before electrochemical testing, both chambers were purged with the feed gas (CO_2 or Ar, 99.99%, Linde Gas) for 10 min to ensure oxygen removal. Subsequently, the outlet ball valve was closed, and the system pressure increased to the desired

value (from 1 to 30 bar). The system was then stirred for 30 minutes to achieve equilibrium, maintaining same pressure in both chambers. For ambient-pressure electrochemical measurements, CO_2 continuously bubbled through the electrolyte at a flow rate of 20 sccm. Potentiostatic tests were performed for 45 minutes with an electrochemical workstation (BioLogic SP-150 Potentiostat). During experiments, the cathode electrolyte was rotated with 380 rpm. Linear Scanning Voltammetry (LSV) were taken with a scan rate of 5 mV s^{-1}. Cathode potentials were converted to the reversible hydrogen electrode (RHE) scale with 70% iR compensation using the following equation:

$$E \text{ (vs RHE)} = E \text{ (vs Ag/AgCl)} + 0.197 + 0.0591 \times pH - iR \times 70\%.$$

Where i represents the current obtained at the corresponding potential, and R is the ohmic resistance of the cell measured at the end of each testing. The iR compensation is to reduce the influence of potentials from the solution resistance on the kinetic model. The pH used in the equation is calculated in the bulk electrolyte under different pressure (The calculation details are shown in the supporting information).

For high pressure conditions, the gas products were collected from an outlet ball valve in a gas bag, and then injected into a gas chromatograph (GC, Trace 1310, Thermo Fisher Scientific). For the ambient pressure condition, gas products flowed into GC directly. Hydrocarbons and carbon monoxide (CO) were detected by a flame ionization detector (FID) and hydrogen (H_2) was quantified by a thermal conductivity detector (TCD). Calibration plots of products are shown in **Figure S5**. Liquid products were quantified via nuclear magnetic resonance (NMR, Bruker, 600-MHZ), using the water suppression method. For NMR analysis, a liquid sample was prepared by mixing 400 μL catholyte with 200 μL internal standard solution (20 ppm DMSO in D_2O). The Faraday efficiency (FE) for each product was expressed as: $FE = \frac{z \times F \times n}{Q}$. Here, z represents the number of electrons transferred, depending on the products, Q is the total charge, n represents the total moles of the product, and F is the Faradaic constant (96,485 C mol^{-1}).

High-pressure in-situ ATR-SEIRAS and Raman spectroscopy

In-situ attenuated total reflectance surface-enhanced infrared absorption spectroscopy (ATR-SEIRAS) were performed for the in-situ monitoring of intermediates on the catalyst surface during the CO_2RR. The ATR crystal was a face-angled Si crystal, which was polished with Al_2O_3 powder and cleaned with deionized water before use. Then, an ultra-thin Au film was sputtered on the surface to enhance the IR signal. The catalyst ink was drop-cast onto this Au film, forming the working electrode. The Si crystal was assembled into a custom-built high-pressure H-cell which was then positioned horizontally in front of an optics system within the chamber of a Nicolet iS50 FT-IR spectrometer for electrochemical ATR-SEIRAS measurements (Tianjin Jike Xingze Technology Co., Ltd.). A Pt foil and an Ag/AgCl electrode served as the counter and reference electrodes, respectively, and a 0.25 M K_2SO_4 solution was used as the electrolyte. High-purity CO_2 was introduced from a gas cylinder to achieve the highest pressure and controllably decreased the pressure through a ball valve and gas regulator (**Figure S22**). The reduction potential was applied using a potentiostat. Spectra are presented in absorption units, defined as $A = -\log(R/R_0)$, where R and R_0 denote the reflected IR intensities of the sample and the reference single-beam spectrum, respectively. The spectra recorded 60 s after application of a new potential. All potentials in in-situ ATR-SEIRAS spectroscopy were converted to the RHE without iR compensation.

The high-pressure Raman cell was made of titanium, equipped with a 3.25 mm sapphire optical window, as previously reported work in our group.[3] The Ag NPs electrode was affixed to the 3D-printed holder using Kapton tape as the working electrode (WE). A Pt foil and an Ag/AgCl electrode were used as the counter and reference electrodes. The pressure inside the Raman cell was controlled by a regulator connected to the CO_2 cylinder, and galvanostatic operation was performed at a fixed current of −10 mA cm^{-2}. In-situ Raman spectra were collected by a confocal micro-Raman spectrometer (WiTec Apyron, Germany) with a 532 nm laser operating at 10 mW.

EIS measurements and DRT analysis

Electrochemical Impedance Spectroscopy (EIS) was measured under CO_2 or Ar pressure of 1 bar to 30 bar by using a potentiostatic operation mode. The frequency range is 100 kHz to 0.1 Hz with 5 points per decade and the amplitude was 10 mV. Each data is the average recorded value of 5 measurements per frequency. The potential was converted to the RHE scale with 70% iR compensation.

The Distribution of relaxation times (DRT) method simulates the impedance of the electrochemistry system including ohmic resistance and an infinite series of polarization resistance. The single polarization process induced by electrochemical/physical processes is described by a RC paralleled circuit consisting of an ohmic resistance R and a capacitance C. Then, kinetic information can be obtained by analyzing the distribution function of relaxation times $g(\tau)$. The relationship between the impedance $Z(\omega)$ and $g(\tau)$ is displayed as:

$$Z(\omega) = R_O + R_P \int_0^{\infty} \frac{g(\tau)}{1+i\omega\tau} d\tau$$

where R_O represents the ohmic resistance, R_P represents the polarization resistance, τ represents the time constant of RC paralleled circuits.

It can be transformed into a logarithmic scale:

$$Z(\omega) = R_O + R_P \int_{-\infty}^{\infty} \frac{\gamma(\ln\tau)}{1+i\omega\tau} d(\ln\tau)$$

Where $\gamma(\ln\tau) = \tau g(\tau)$

The resistance of each RC component in the equivalent circuit can be determined by integrating lnτ:

$$R_P = \int_{\tau_l}^{\tau_u} \gamma(\ln\tau) d(\ln\tau)$$

However, the calculation of $g(\tau)$ is sensitive to data noise and is associated with ill-posed problems. Therefore, Tikhonov regularization was implemented to adjust for noise resistance. It means adding a penalty function P(x) to the objective function to reduce DRT oscillation as shown below:

$$S(x) = \|Z'_{DRT} - Z'_{exp}\|^2 + \|Z''_{DRT} - Z''_{exp}\|^2 + \lambda P(x)$$

Where $\|Z'_{DRT} - Z'_{exp}\|^2 + \|Z''_{DRT} - Z''_{exp}\|^2$ is the fit residual term; $\lambda P(x)$ is the regularization term. By minimizing the $\lambda P(x)$, the regularization parameter λ can be adjusted to control the oscillation of DRT. The residuals between the measured impedance data and the impedance obtained from DRT were calculated at different λ values[4]:

$$\sigma_{re} = \frac{x_{re,s} - x_{re,\omega}}{|x_\omega|}$$

$$\sigma_{im} = \frac{x_{im,s} - x_{im,\omega}}{|x_\omega|}$$

Where $x_{re,s}$ and $x_{im,s}$ are the real and imaginary values of impedance from DRT respectively, $x_{re,\omega}$ and $x_{im,\omega}$ are the actual measured real and imaginary values. $|x_\omega|$ is the absolute value of the actual impedance. The sum of squared residuals (SSR) between the measured impedance data and data reconstructed from DRT was further calculated. The impedance measured at −0.95 V vs RHE under 1–30 bar was used for optimizing regularization parameter λ. The DRT analysis was developed using the DRT Tools in MATLAB software.[5-7]

Calculation of bulk and local concentrations of carbon species and corresponding pH

When dissolved in the electrolyte, gaseous CO_2 undergoes a series of chemical reactions and reaches the equilibrium:

$$CO_2(aq) + H_2O \rightleftharpoons H_2CO_3;\ K_1 = 2.63 \times 10^{-3}\ M$$

$$H_2CO_3 \rightleftharpoons H^+ + HCO_3^-;\ K_2 = 1.7 \times 10^{-4}\ M$$

It is assumed that $[H_2CO_3]$ is in equilibrium with $[CO_2(aq)]$ and the sum is represented as CO_2^*:

$$[CO_2^*] = [CO_2(aq)] + [H_2CO_3]$$

The dissociation of H_2CO_3 could be further written as:

$$CO_2^* + H_2O \rightleftharpoons H^+ + HCO_3^-;\ K_3 = 4.45 \times 10^{-7}\ M$$

The second step of dissociation and H_2O ionization equilibrium:

$$HCO_3^- \rightleftharpoons H^+ + CO_3^{2-};\ K_4 = 4.84 \times 10^{-11}\ M$$

$$H_2O \rightleftharpoons H^+ + OH^-;\ K_5 = 1 \times 10^{-14}\ M^2$$

According to Henry's law, the partial pressure of CO_2 in the gas phase is directly proportional to the concentration of dissolved CO_2 at equilibrium[8]:

$$[CO_2(aq)] = k_h \cdot P_{CO_2};\ k_h = 0.034\ \text{mol/(kg·bar)}$$

According to charge conservation in electrolyte:

$$[K^+] + [H^+] = [OH^-] + [HCO_3^-] + 2[CO_3^{2-}]$$

The bulk concentrations of carbon species (e.g., CO_2; HCO_3^-; CO_3^{2-}) and pH in 0.5 M $KHCO_3$ electrolyte under CO_2 pressure from 1 bar to 30 bar were calculated using MATLAB.

For local concentration calculation, when current is applied, CO_2 and other carbon species diffuse from the bulk electrolyte to the electrode surface. Simultaneously, reaction products desorb from the electrode and diffuse in the opposite direction, establishing a diffusion layer with a concentration gradient (**Figure S15**).[8, 9] Because of the higher pH in this layer than that in the bulk electrolyte, CO_2 directly reacts with OH^- and forms HCO_3^-:

$$CO_2 + OH^- \rightleftharpoons HCO_3^-$$

$$HCO_3^- + OH^- \rightleftharpoons H_2O + CO_3^{2-}$$

The local concentration of these species could be calculated via four partial differential equations (PDEs) and two boundary conditions by using *pdepe* library in MATLAB.:

$$\frac{\partial[CO_2]}{\partial t} = D_{CO_2}\frac{\partial^2[CO_2]}{\partial x^2} + k_{-1}[HCO_3^-] - k_1[CO_2][OH^-]$$

$$\frac{\partial[HCO_3^-]}{\partial t} = D_{HCO_3^-}\frac{\partial^2[HCO_3^-]}{\partial x^2} + k_1[CO_2][OH^-] + k_{-2}[H_2O][CO_3^{2-}] - k_{-1}[HCO_3^-] - k_2[HCO_3^-][OH^-]$$

$$\frac{\partial[CO_3^{2-}]}{\partial t} = D_{CO_3^{2-}}\frac{\partial^2[CO_3^{2-}]}{\partial x^2} + k_2[HCO_3^-][OH^-] - k_{-2}[H_2O][CO_3^{2-}]$$

$$\frac{\partial[OH^-]}{\partial t} = D_{OH^-}\frac{\partial^2[OH^-]}{\partial x^2} + k_{-1}[HCO_3^-] + k_{-2}[H_2O]\,[CO_3^{2-}] - k_1[CO_2]\,[OH^-] - k_2[HCO_3^-]\,[OH^-]$$

where the t and x are time and position in the diffusion layer. k_n and k_{-n} represents the forward and backward rate constants The values of the diffusion coefficient (D) and rate constants (k_n/k_{-n}) are shown in **Table S2**. The diffusion layer thickness (δ) was set to 50 μm.[9] This thickness is assumed to remain constant with increasing pressure in simulations because liquid water is incompressible in 1−30 bar. Therefore, the liquid density and viscosity change only slightly, and the change in δ is negligible.

Two boundary conditions: At x = 0; the concentrations of these species (CO_2; HCO_3^-; CO_3^{2-}; OH^-) are equal to that in the bulk electrolyte;

At x = δ (the electrode-electrolyte interface):

$$D_{CO_2}\frac{\partial[CO_2]}{\partial x} = \text{-Consumption rate of } [CO_2] = -\frac{j_{total}}{F}\left(\frac{FE_{CO}}{2} + \frac{FE_{HCOO^-}}{2}\right)$$

$$D_{HCO_3^-}\frac{\partial[HCO_3^-]}{\partial x} = 0$$

$$D_{CO_3^{2-}}\frac{\partial[CO_3^{2-}]}{\partial x} = 0$$

$$D_{OH^-}\frac{\partial[OH^-]}{\partial x} = \text{Generation rate of } [OH^-] = \frac{j_{total}}{F}$$

Rate expression and Tafel slope calculation

For electrochemical reaction (O and R represent oxidant and reductant, respectively):

$$O + ne^- \rightarrow R$$

The Butler-Volmer equation is typically employed, and the simplified rate expression is shown as:

$$j = -nFka[O]\exp(-\alpha f\eta)$$

j is the current density; n is the number of transferred electrons;

η is the activation overpotential; it is the difference between the applied voltage and the equilibrium voltage (E^0); For two reactions[10]:

$2H^+ + 2e^- \leftrightarrow H_2$ ($E^0 = 0V$)

$CO_2 + 2H^+ + 2e^- \leftrightarrow CO + H_2O$ ($E^0 = -0.1V$)

E^0 is the equilibrium potential at a standard condition. k is the rate constant; α is the transfer coefficient; a[O] is the concentration of oxidant; f = F/RT, where F is the Faraday constant, R is the ideal gas constant and T is absolute temperature.[11, 12]

The elementary steps of mechanism 1 and 2 are shown in **Table 1**. We take mechanism 2 as an example to deduce the rate expression and Tafel slope.

If the first step is assumed as the rate-limiting step (RLS), the rate expression is:

$$j_{CO} = -2Fk_{B1}^0 a_{CO_2} a_{H_2O}\theta\exp(-\alpha f\eta)$$

According to the Tafel formula, η = A + B log(j), the Tafel slope is:

$$\frac{\partial(-\eta)}{\partial(\log[j_{CO}])} = \frac{1}{\alpha f\log e}$$

If the second step is assumed as the RLS, the rate expression is:

$$j_{CO} = -2Fk_{B2}^0[*\,COOH]\exp(-\alpha f\eta)$$

And the step before the RLS is in fast equilibrium:

$$k_{B1}^0 a_{CO_2} a_{H_2O} \theta \exp(-\alpha f \eta) = k_{-B1}^0 [*\mathrm{COOH}] a_{OH^-} \exp[(1-\alpha) f \eta];$$

$$[*\mathrm{COOH}] = \frac{K_{B1}^0 a_{CO_2} a_{H_2O} \theta \exp(-f\eta)}{a_{OH^-}}$$

Where K^0 is the standard equilibrium constant, $K^{B1} = k^{B1}/k_{-B1}^0$; k^{B1}, k_{-B1}^0 are the forward and reverse rate constants respectively.

The rate expression and corresponding Tafel slope can be deduced:

$$j = -\frac{2Fk_{B2}^0 K_{B1}^0 a_{CO_2} a_{H_2O} \theta \exp(-(1+\alpha) f\eta)}{a_{OH^-}}$$

$$\frac{\partial(-\eta)}{\partial(\log[j_{CO}])} = \frac{1}{(1+\alpha) f \log e}$$

If the third step is assumed as the RLS, the rate expression and corresponding Tafel slope can be deduced:

$$j_{CO} = -2Fk_{B3}^0 [*\mathrm{CO}]$$

$$[*CO] = \frac{K_{B1}^0 K_{B2}^0 a_{CO_2} a_{H_2O} \theta \exp(-2f\eta)}{a_{OH^-}^2}$$

$$j = -\frac{2Fk_{B3}^0 K_{B1}^0 K_{B2}^0 a_{CO_2} a_{H_2O} \theta \exp(-2f\eta)}{a_{OH^-}^2}$$

$$\frac{\partial(-\eta)}{\partial(\log[j_{CO}])} = \frac{1}{2\alpha f \log e}$$

Kinetic model

As CO and H_2 are the major products, the formate pathway is not considered in this study. All the reaction steps are considered reversible, and the rate expression of step 1-5 is given as:

$$r_1 = r_{1f} - r_{1b} = k_1 \theta_1 a_{CO_2} a_{H_2O} - k_{-1} \theta_2 a_{OH^-} \quad (1)$$

$$r_2 = r_{2f} - r_{2b} = k_2 \theta_2 - k_{-2} \theta_3 a_{OH^-} \quad (2)$$

$$r_3 = k_3 \theta_3 \quad (3)$$

$$r_4 = r_{4f} - r_{4b} = k_4 \theta_1 a_{H_2O} - k_{-4} \theta_4 a_{OH^-} \quad (4)$$

$$r_5 = k_5 \theta_4 a_{H_2O} \quad (5)$$

Where r_{nf} and r_{nb} are the forward and backward rate of step n respectively; θ_1 = bare coverage; θ_2 = coverage of *COOH; θ_3 = coverage of *CO; θ_4 = coverage of *H. The relevant concentration of CO_2 and OH^- is determined from the mass transport model as described in the former section. k_n and k_{-n} are the rate constants of the forward and reverse reactions of step n respectively, are shown as:

$$k_n = k_n^0 \exp(-\alpha_n f \eta); \; k_{-n} = k_{-n}^0 \exp[(1-\alpha_n) f \eta]$$

Where k_n^0 and k_{-n}^0 are the standard rate constant. α_n is the transfer coefficient. For step 3, $k_3 = k_3^0$ as it does not involve electron transfer.

The surface coverages of adsorbed species (*COOH; *CO; *H) are assumed in the steady-state which means their formation rate is equal to their consumption rate. The total coverage is equal to 1.

$$\frac{d\theta_2}{dt} = r_1 - r_{-1} - r_2 + r_{-2} = 0$$

$$\frac{d\theta_3}{dt} = r_2 - r_{-2} - r_3 = 0$$

$$\frac{d\theta_4}{dt} = r_4 - r_{-4} - r_5 = 0$$

$$\theta_1 + \theta_2 + \theta_3 + \theta_4 = 1$$

The coverage expression can be derived as following:

$$\theta_2 = \frac{k_1 a_{CO_2} a_{H_2O}[k_3 + k_{-2} a_{OH^-}]}{k_2 k_3 + k_{-1} k_3 a_{OH^-} + k_{-1} k_{-2} a_{OH^-}^2} \theta_1$$

$$\theta_3 = \frac{k_1 k_2 a_{CO_2} a_{H_2O}}{k_2 k_3 + k_{-1} k_3 a_{OH^-} + k_{-1} k_{-2} a_{OH^-}^2} \theta_1$$

$$\theta_4 = \frac{k_4 a_{H_2O}}{k_{-4} a_{OH^-} + k_5 a_{H_2O}} \theta_1$$

$$\theta_1 = \frac{1}{\frac{k_4 a_{H_2O}}{k_{-4} a_{OH^-} + k_5 a_{H_2O}} + \frac{k_1 a_{CO_2} a_{H_2O}[k_2 + k_3 + k_{-2} a_{OH^-}]}{k_2 k_3 + k_{-1} k_3 a_{OH^-} + k_{-1} k_{-2} a_{OH^-}^2} + 1}$$

The partial current densities of CO and H_2 in kinetic model are given as:

$$j_{\text{CO-model}} = -F(r_1 + r_2)$$

$$j_{\text{H2-model}} = -F(r_4 + r_5)$$

Before calculation, the partial current densities of reaction products in the range of −1.1 V to −1.6 V vs RHE were interpolated based on the potentiostatic test results. **Figure S18** shows the experimental results and the line represents the corresponding interpolated (B-Spline) results.[13] The calculation is based on Global optimization with lsqnonlin method in MATLAB, which was used to find optimal solutions for objective function f(x) with minimum residual:

$$f(x) = \begin{bmatrix} w1 \times (\log[j_{\text{CO-model}}] - \log[j_{\text{CO-exp}}]) \\ w2 \times (\log[j_{\text{H2-model}}] - \log[j_{\text{H2-exp}}]) \end{bmatrix}$$

Where W1 and W2 are the weight of two reactions in the objective function. W1=0.8, W2 =0.2 was set. Some parameters for computation and constraints for k^0 and α are shown in **Table S5**.

Kinetic model for CO_2-to-formate

The kinetic modeling framework was further extended to our previous work of CO_2 electroreduction to formate on liquid metals, which used the same high-pressure experimental condition as in the present work.[3] All the reaction steps are considered reversible, and the adsorbed *CO_2 in step B1 is in quasi-equilibrium with their bulk concentrations as the rate of adsorption and desorption are relatively faster than that of surface reactions. The rate expression of step B2-3 is given as:

$$r_{B2} = r_{B2f} - r_{B2b} = k_{B2}\theta_{B2} a_{H_2O} - k_{-B2}\theta_{B3} a_{OH^-}$$

$$r_{B3} = r_{B3f} - r_{B3b} = k_{B3}\theta_3 a_{H_2O} - k_{-B3}\theta_{B4} a_{OH^-}$$

$$r_{B4} = k_{B4}\theta_{B4}$$

Where θ_{B1} = bare coverage; θ_{B2} = coverage of *CO_2; θ_{B3} = coverage of *HCOO; θ_{B4} = coverage of *HCOOH; θ_{B5} = coverage of *H. The coverage expression can be derived as following:

$$\theta_{B2} = K_{B1} a_{CO_2} \theta_{B1}$$

$$\theta_{B3} = \frac{k_{B2} K_{B1} a_{CO_2} a_{H_2O}(k_{-B3} a_{OH^-} + k_{B4})\theta_1}{k_{B3} k_{B4} a_{H_2O} + k_{-B2} k_{-B3} a_{OH^-}^2 + k_{-B2} k_{B4} a_{OH^-}}$$

$$\theta_{B4} = \frac{k_{B3} a_{H_2O} \theta_{B3}}{k_{-B3} a_{OH^-} + k_{B4}}$$

$$\theta_{B5} = \frac{k_{B5} a_{H_2O} \theta_{B1}}{k_{-B5} a_{OH^-} + k_{B6} a_{H_2O}}$$

$$\theta_{B1} = \frac{1}{\frac{k_{B5}a_{\mathrm{H_2O}}}{k_{-B5}a_{\mathrm{OH^-}} + k_{B6}a_{\mathrm{H_2O}}} + K_{B1}a_{\mathrm{CO_2}} + \frac{k_{B2}K_{B1}a_{\mathrm{CO_2}}a_{\mathrm{H_2O}}\left(k_{B3}a_{\mathrm{H_2O}} + k_{-B3}a_{\mathrm{OH^-}} + k_{B4}\right)}{k_{-B2}k_{B4}a_{\mathrm{OH^-}} + k_{-B2}k_{-B3}a_{\mathrm{OH^-}}^2 + k_{B3}k_{B4}a_{\mathrm{H_2O}}} + 1}$$

K_{B1} is the equilibrium constant for step 1; k_{Bn} and k_{-Bn} are the rate constants of the forward and reverse reactions of step n respectively, are shown as:

$$k_{Bn} = k_{Bn}^0\exp(-\alpha_n f\eta);\ k_{-Bn} = k_{-Bn}^0\exp[(1-\alpha_n)f\eta]$$

The equilibrium voltage for the reaction:

$CO_2 + 2H^+ + 2e^- \leftrightarrow HCOOH$ ($E^0 = -0.2V$)

The partial current densities of formate in kinetic model are given as:

$$j_{\text{HCOOH-model}} = -F(r_{B2} + r_{B3})$$

The parameters for computation and constraints for k^0 and α are shown in **Table S7** and **Table S8**.

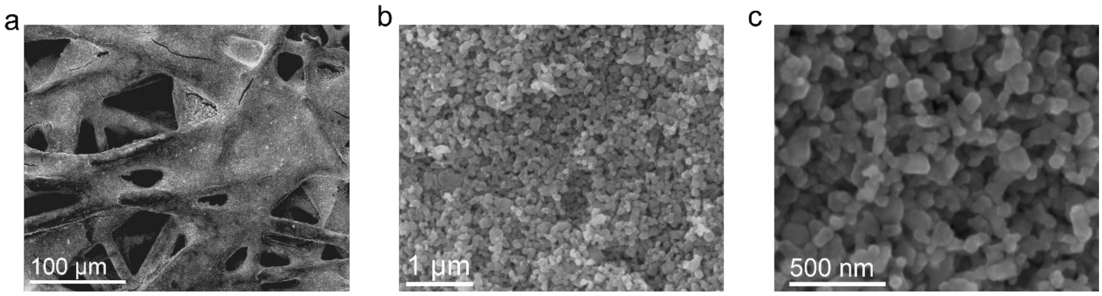

Figure S1. SEM images of Ag NPs sprayed on the carbon paper at various magnifications.

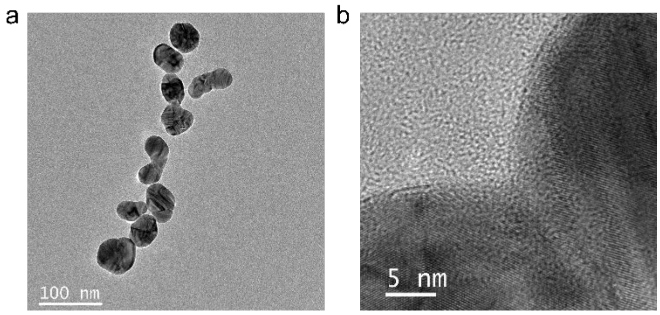

Figure S2. TEM images of Ag NPs at low (a) and high magnifications (b).

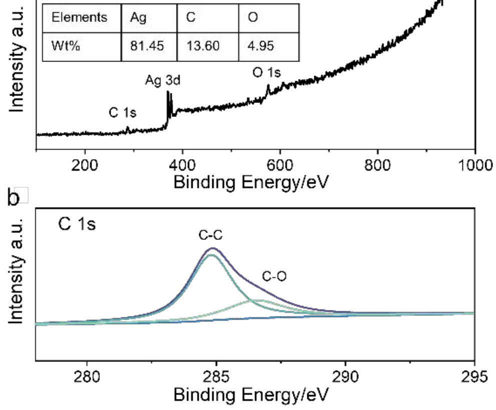

Figure S3. XPS spectra of Ag NPs: (a) survey, (b) C 1s spectra. Insert: weight percentage of Ag,

C, O in Ag NPs.

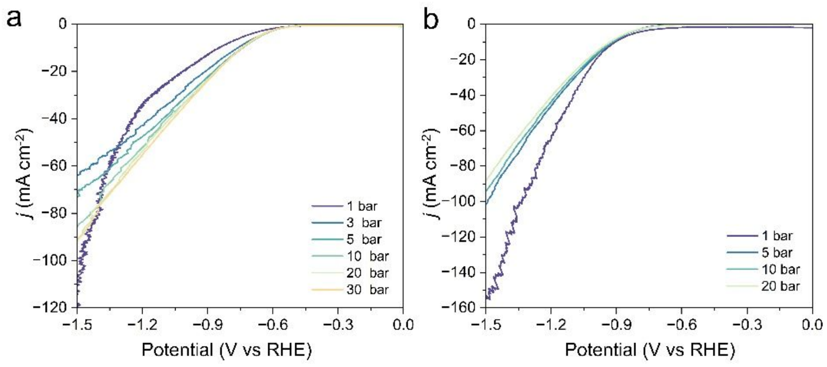

Figure S4. (a) LSV curves in 0.5 M $KHCO_3$ under 1−30 bar of CO_2 (scan rate: 5 mV s^{-1}). (b) LSV curves under 1−20 bar of Ar (scan rate: 5 mV s^{-1}).

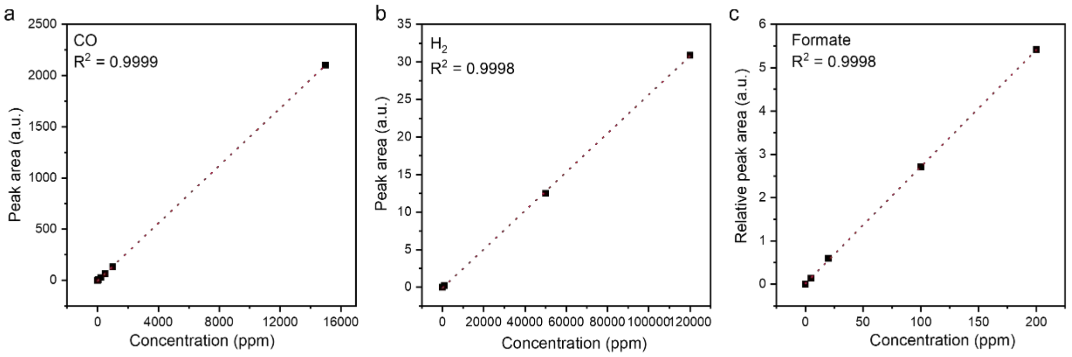

Figure S5. Calibration curves of CO (a), H_2 (b) and formate (c).

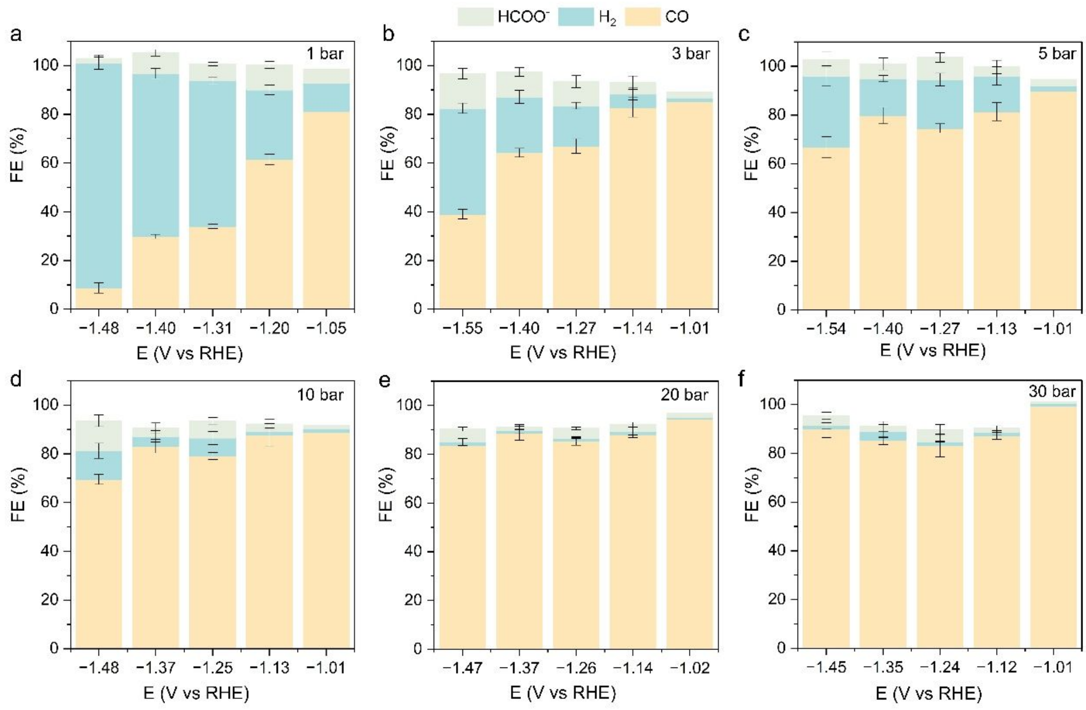

Figure S6. Products distribution over Ag NPs at various potentials and CO_2 pressures.

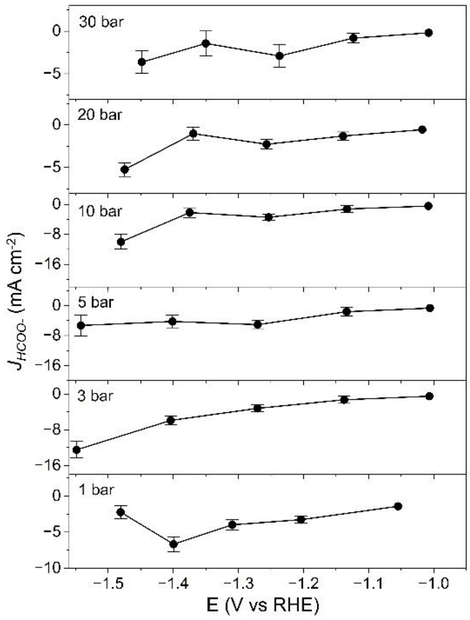

Figure S7. Partial current densities of formate under different CO_2 pressures and potentials.

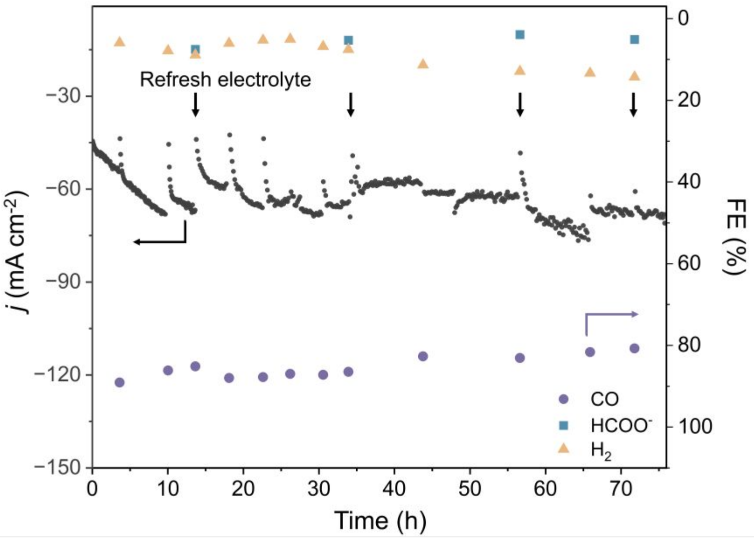

Figure S8. Chronoamperometry test of Ag NPs at –1.46 V vs RHE under 30 bar without iR compensation. Positions indicated by black arrows represent the replacement of the electrolyte.

It should be noted that product analysis in the high-pressure H-cell was conducted offline rather

than in real-time. Gas products were collected in gas bags by depressurizing the system to 1 bar. Consequently, the stability test was periodically interrupted (12 intervals), followed by re-pressurization with CO_2 to 30 bar and a 10-minute equilibration period before continuing the test. Similarly, liquid products were collected 4 times and the electrolyte was refreshed after each collection (shown as black arrows in **Figure S8**).

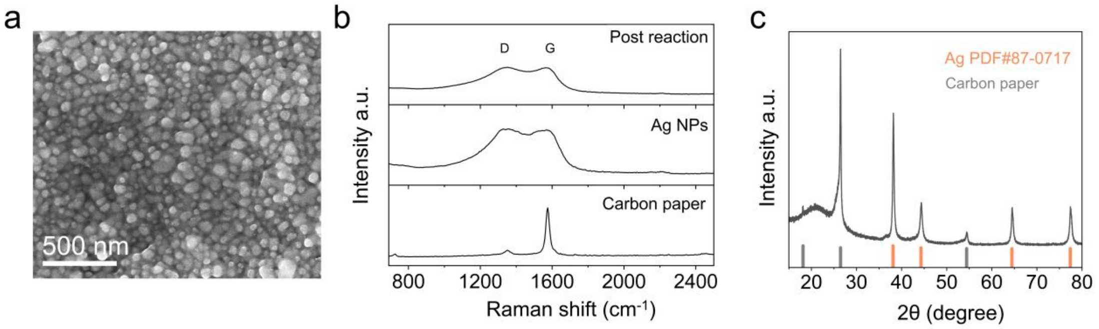

Figure S9. (a) SEM image, (b) Raman spectra, and (c) XRD pattern of the electrode after the stability test. In the Raman spectra, the I_D/I_G ratio of the PVP layer is distinct from that of the carbon paper substrate. In the XRD pattern, the gray bar chart corresponds to the bare carbon paper substrate.

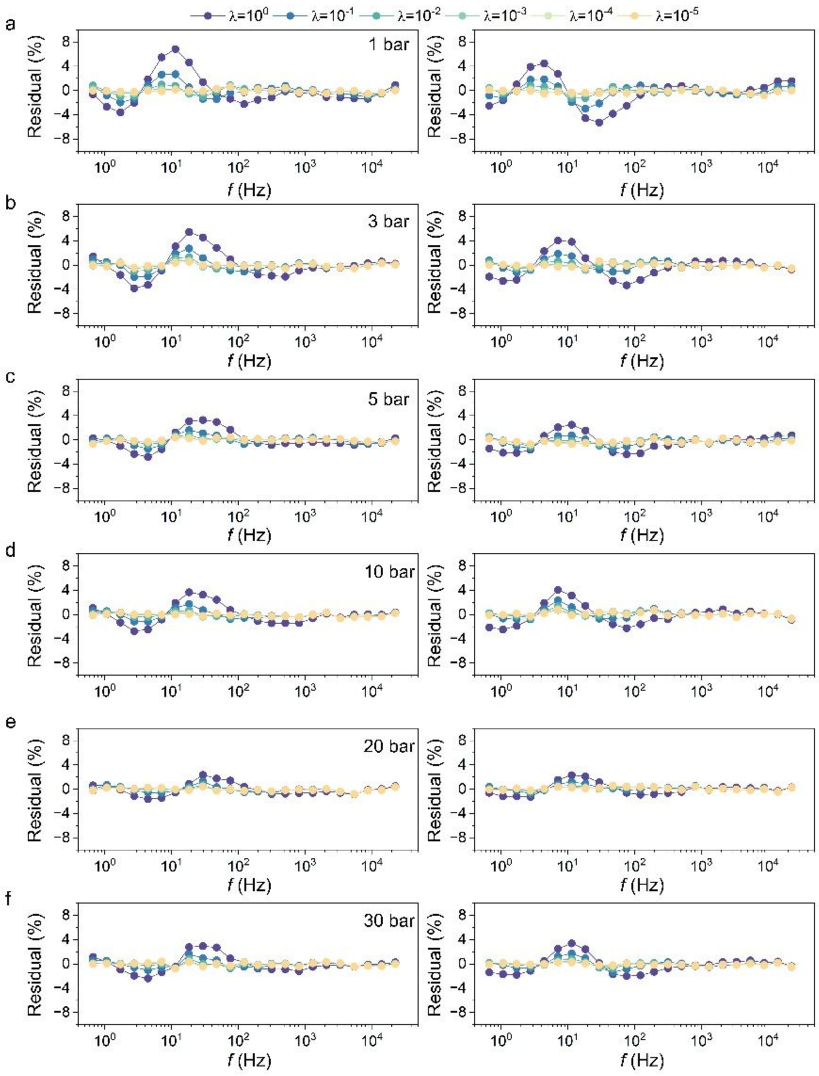

Figure S10. The residual analysis of real part (left) and imaginary part (right) of different regularization parameters λ under 1−30 bar.

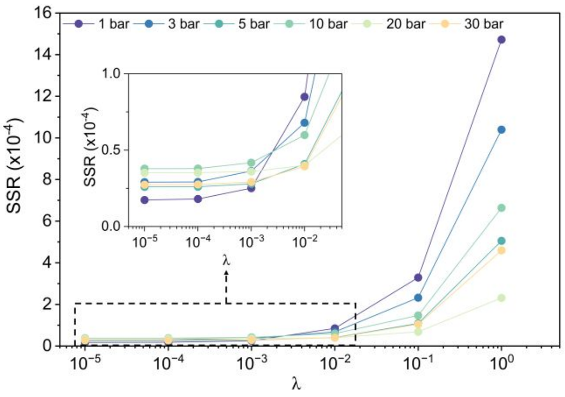

Figure S11. SSR calculated from residuals as a function of λ.

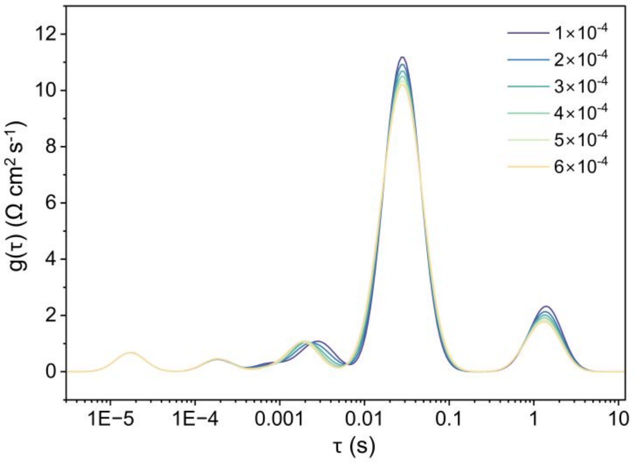

Figure S12. The DRT spectra for λ values from 1×10^{-4} to 6×10^{-4}.

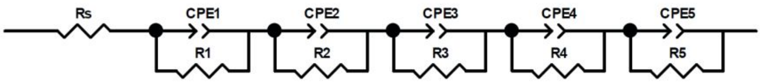

Figure S13. The corresponding equivalent circuit model, including one ohmic resistance (Rs) and five series-connected RC components.

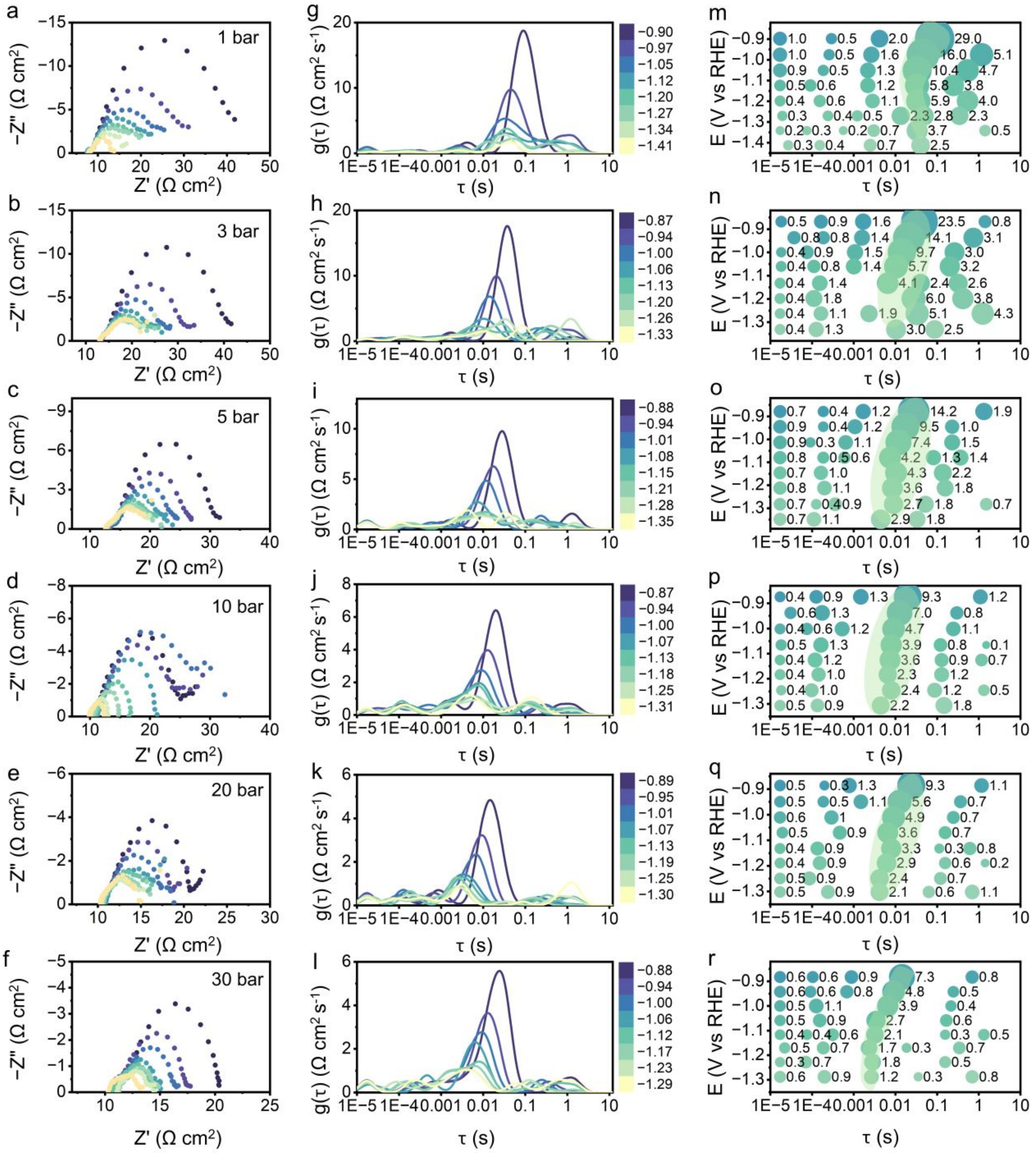

Figure S14. EIS spectra for CO_2 reduction under 1−30 bar (a-f). (g-l) the corresponding DRT plots. The scale bar shows the potential range under the respective pressures (unit: V vs. RHE). polarization resistance derived from the respective DRT plots. The scale bar shows the value of polarization resistance (Unit: Ω cm^2).

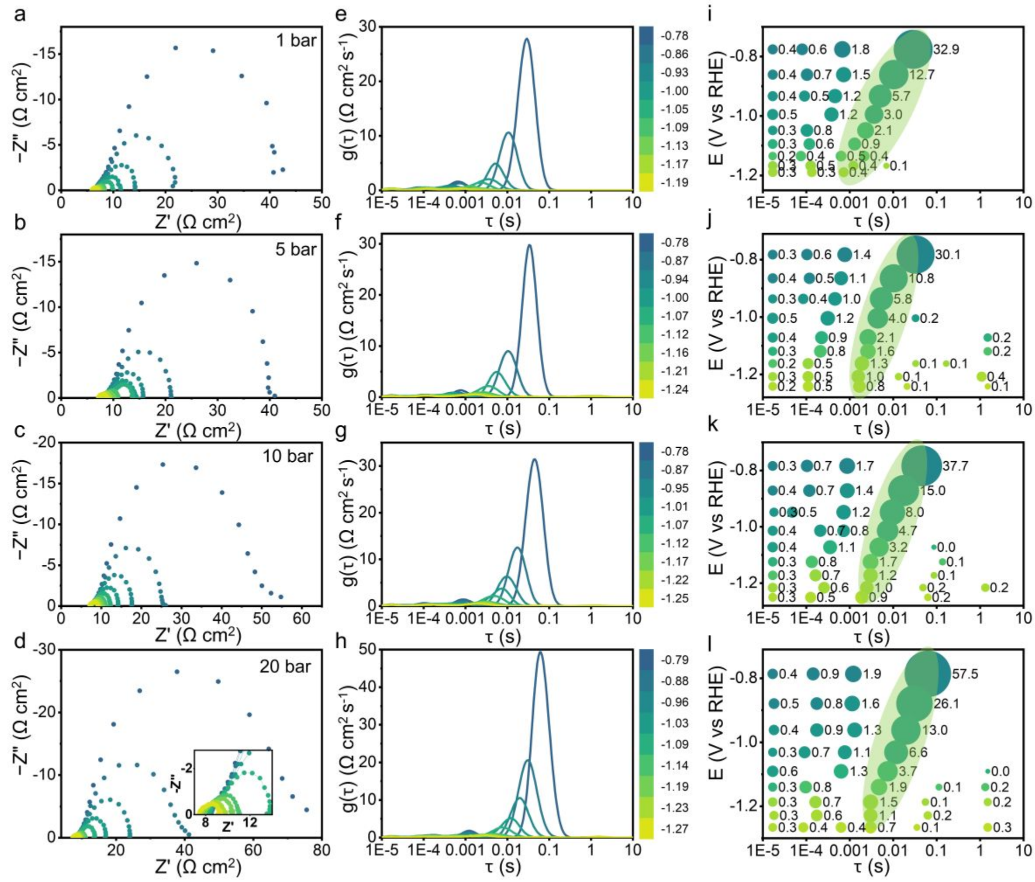

Figure S15. EIS spectra for HER under (a) 1 bar, (b) 5 bar, (c) 10 bar and (d) 20 bar. (e-h) the corresponding DRT plots. The scale bar shows the potential range (unit: V vs. RHE). (i-l) polarization resistance derived from the respective DRT plots. The scale bar shows the value of polarization resistance (Unit: Ω cm^2).

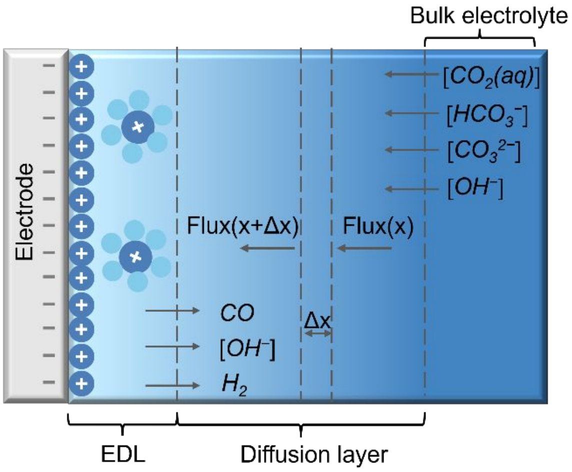

Figure S16. Diffusion–reaction model for CO_2RR and HER in the diffusion layer.

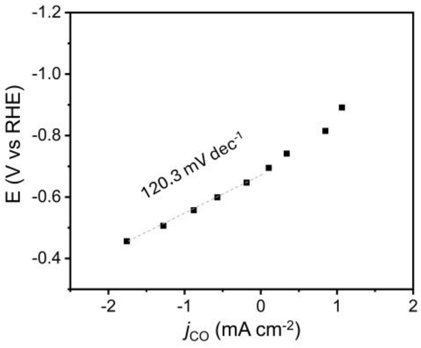

Figure S17. Tafel plots under 1 bar of CO_2.

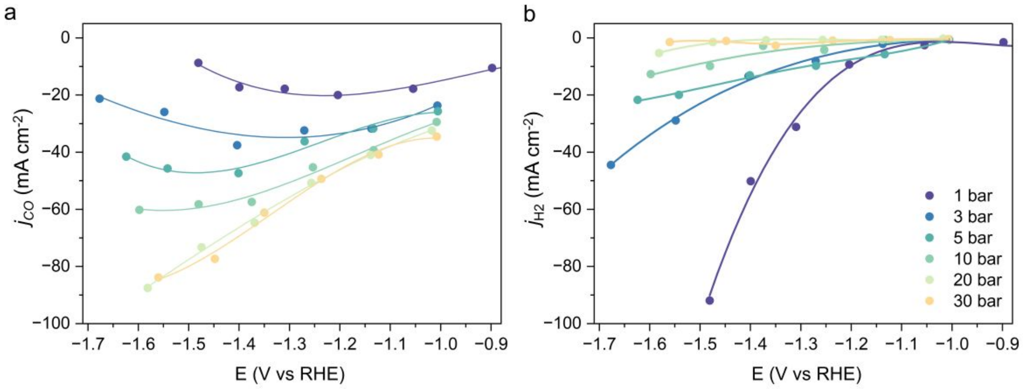

Figure S18. The partial current densities of CO (a) and H_2 (b) under pressure of 1-30 bar and potential range of −1.0 V to −1.7 V vs RHE. The line represents the corresponding interpolated (B-Spline) results.

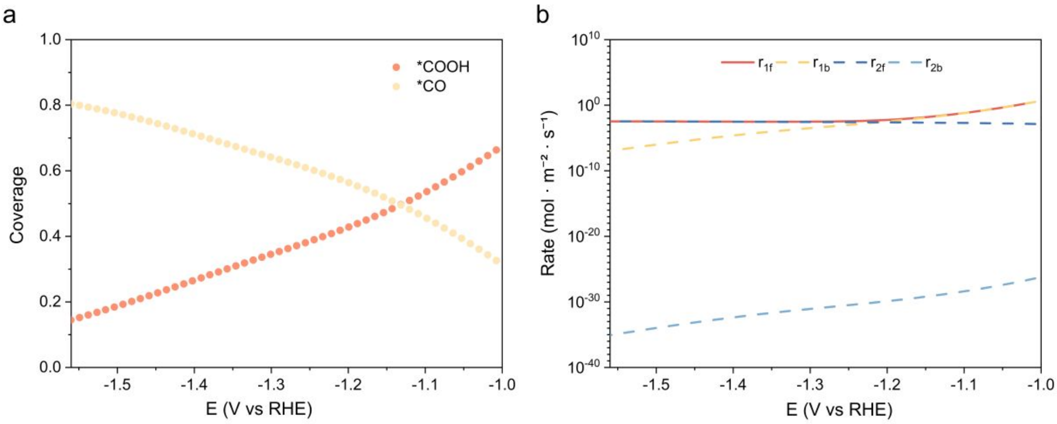

Figure S19. (a) Simulated surface coverages of *COOH and *CO as a function of potential at 30 bar. (b) Steady-state forward and reverse reaction rates of step 1 and step 2 at 30 bar.

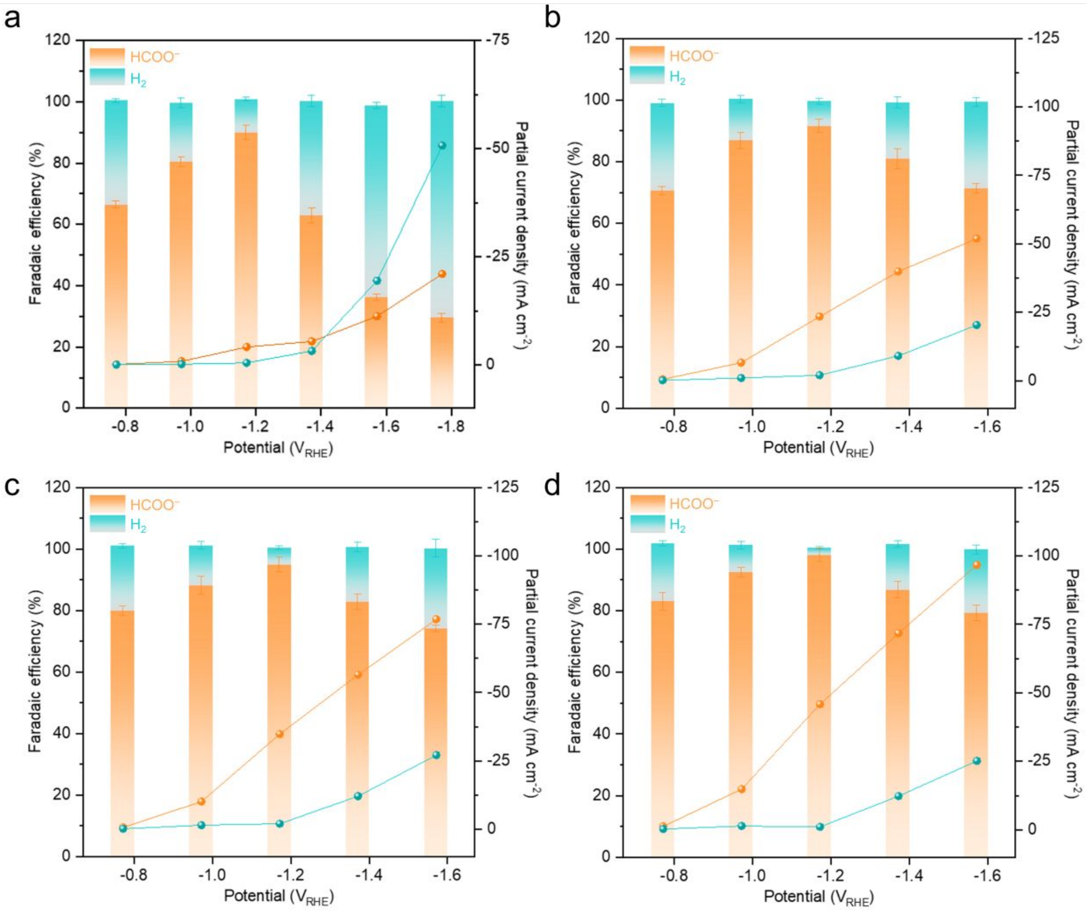

Figure S20. The partial current densities of $HCOO^-$ and H_2 on S47 under pressure of 1 bar (a), 10 bar (b), 20 bar (c) and 30 bar (d) at potential range of −0.8 V to −1.6 V vs RHE.

Reprinted with permission from J. Zhao, M. Jin, H. Huang, H. Guo, Y. Han, L. Huang, Y.-Y. Yuan, Q. Wang, J. Liang, X. Gao, J. Long, N. Tsubaki, X. Lu, Unlocking atomic degrees of freedom in liquid metals for accelerated electrocatalytic reactions, ACS Catalysis 2025, 15, 3505-3514. DOI: 10.1021/acscatal.5c00087 Copyright [2025] [American Chemical Society].

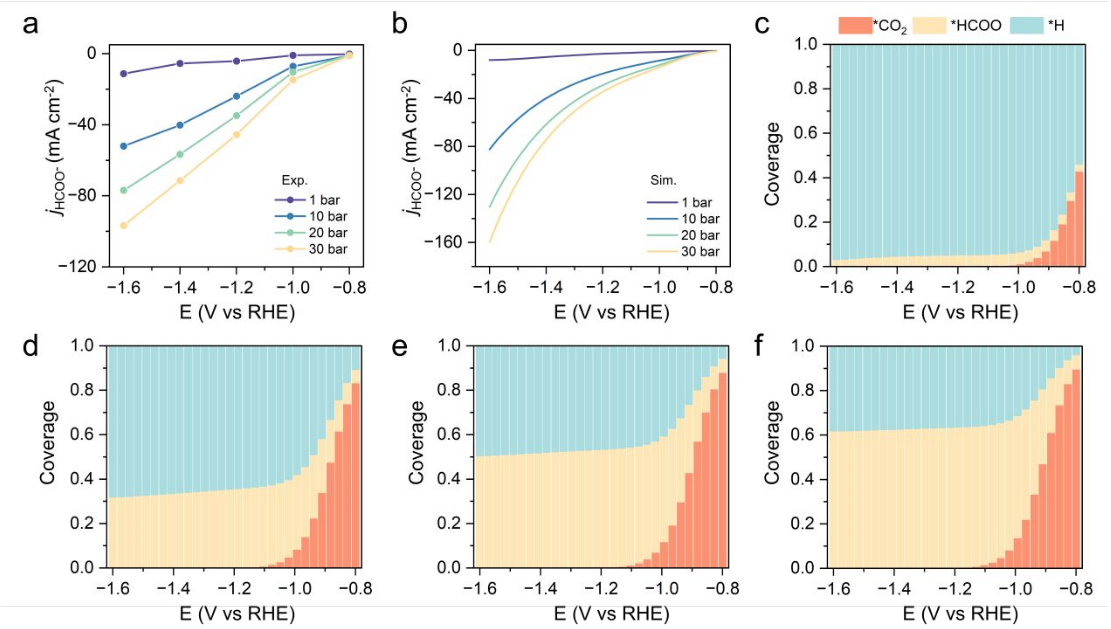

Figure S21. The partial current densities of $HCOO^-$ from experimental data (a) and simulation (b) under pressure of 1−30 bar. The predicted fractional surface coverage of $*CO_2$, *HCOO and *H under 1 bar (c), 10 bar (d), 20 bar (e), 30 bar (f).

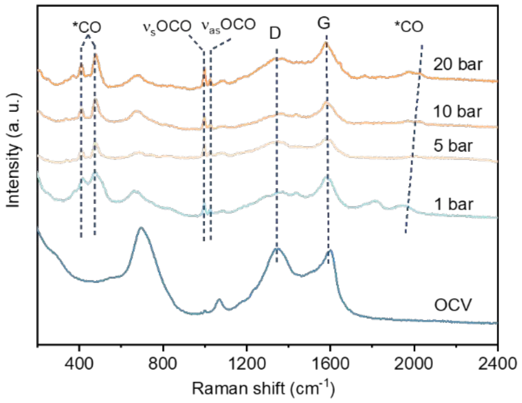

Figure S22. Pressure-dependent In-situ Raman spectra acquired on the Ag NPs surface at the open-circuit potential and a constant current density of −10 mA cm^{-2}.

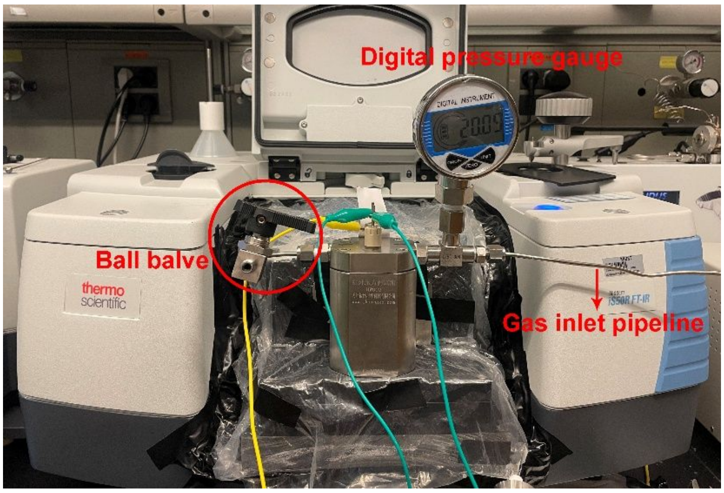

Figure S23. Photograph of High-pressure in-situ ATR-SEIRAS apparatus. Digital pressure gauge shows the operating pressure.

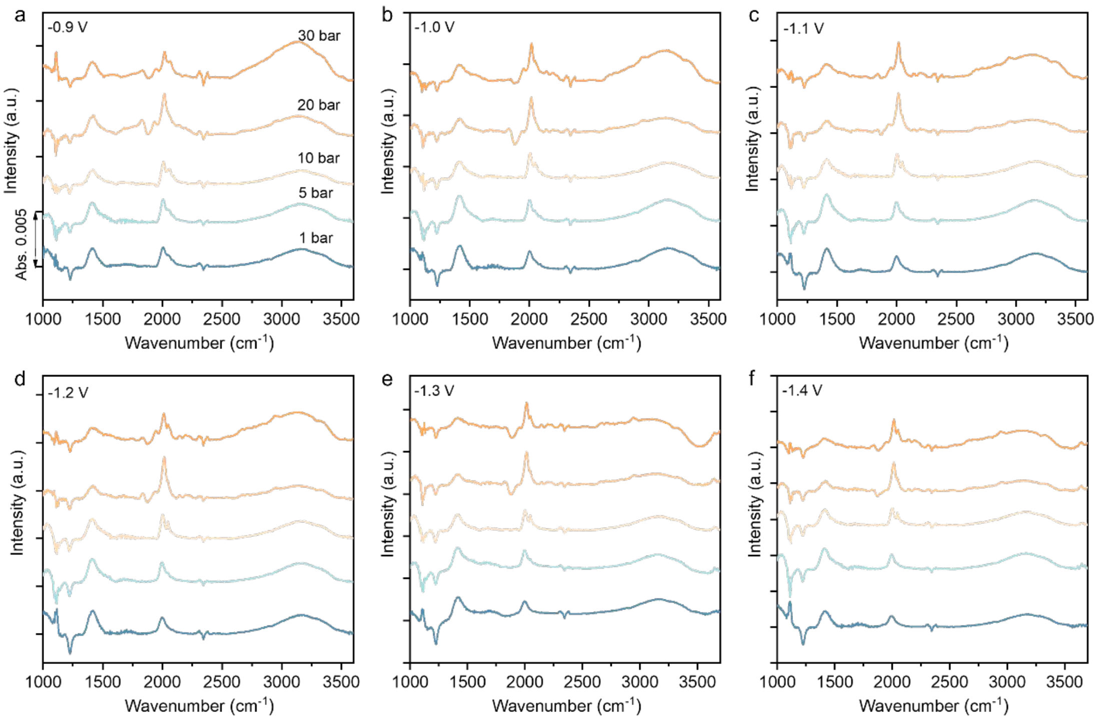

Figure S24. Pressure-dependent In-situ ATR-SEIRA spectra acquired on the Ag NPs surface at potentials of −0.9 V to −1.4 V vs RHE.

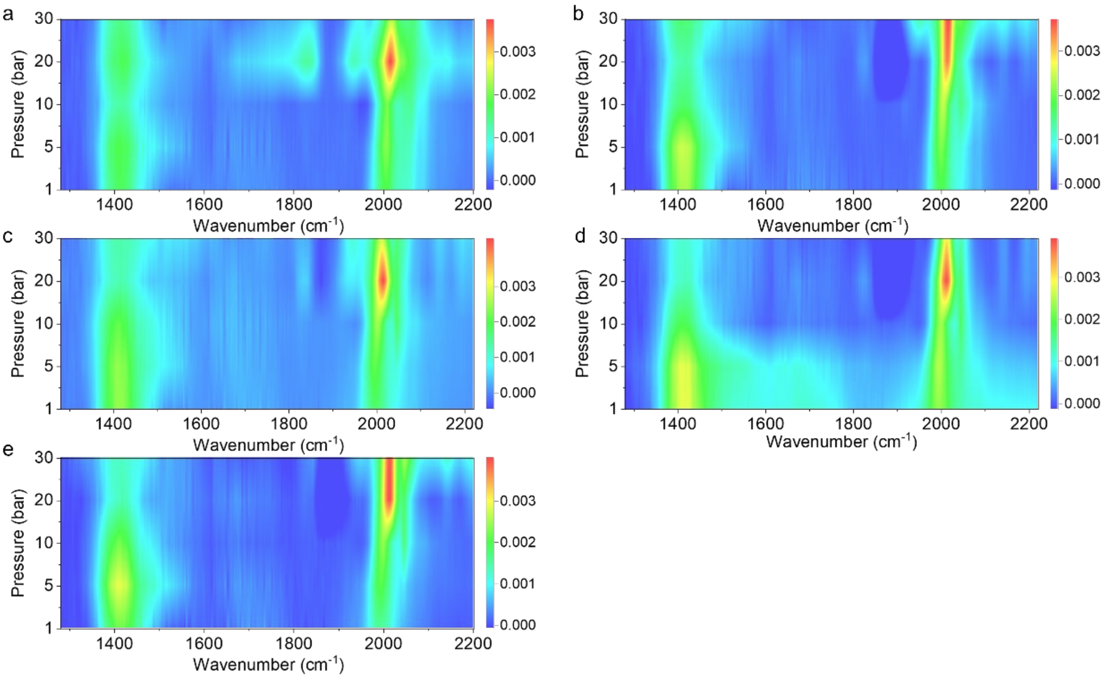

Figure S25. Corresponding heat map of ATR-SEIRA spectra as a function of pressure (1-30 bar) at the potential of (a) −0.9 V, (b) −1.0 V, (c) −1.1 V, (d) −1.3 V and (e) −1.4 V. All potentials vs RHE.

Table S1. Impedance analysis and DRT peak assignment for CO_2RR.

Peak	Time constant, τ (s)	Assignment
1,2	10^{-5}–10^{-3}	Ion transfer process
3	10^{-3}–10^{-1}	CO_2RR charge transfer process
4	10^{-3}–10^{-1}	Rate-limiting step of CO_2RR
5	10^{-1}–10	CO_2 mass transfer process

Table S2. Values of diffusion coefficient and rate constants.

Diffusivity (m^2 s^{-1})	Rate constant
$\boldsymbol{D}_{CO_2} = 1.91 \times 10^{-9}$	$k_1 = 5.93 \times 10^3$ (M^{-1} S^{-1})
$\boldsymbol{D}_{HCO_3^-} = 9.23 \times 10^{-10}$	$k_{-1} = 1.34 \times 10^{-4}$ (S^{-1})
$\mathbf{D}_{CO_3^{2-}} = 1.19 \times 10^{-9}$	$k_2 = 1 \times 10^8$ (M^{-1} S^{-1})
$\boldsymbol{D}_{OH^-} = 5.27 \times 10^{-9}$	$k_{-2} = 2.15 \times 10^4$ (S^{-1})

Table S3. Parameter for lsqnonlin method.

Algorithm	Trust-region-reflective
MaxIterations	10^4
MaxFunctionEvaluations	5×10^5
OptimalityTolerance	10^{-6}
StepTolerance	10^{-6}

Table S4. Constraints for k^0 and α.

	Lower bound	Upper bound
k^0	10^{-13}	10^{15}
α	0.1	0.5

Table S5. The evaluation parameters of the accuracy of the kinetic model simulation.

Parameters	Value
Root Mean Squared Error (RMSE)	0.2715
Mean Absolute Error (MAE)	0.2326
Coefficient of Determination (R^2)	0.9911
Relative Error (RE%)	13.7773%
Pearson Correlation Coefficient (r)	0.9956

Table S6. Best-Fitted kinetic parameters from the kinetic model.

Kinetic parameters	Value
$\boldsymbol{k_1^0}$	4.7×10^{10}
$\boldsymbol{k_{-1}^0}$	1.8×10^{11}
$\boldsymbol{k_2^0}$	9.8×10^{-5}
$\boldsymbol{k_{-2}^0}$	3.0×10^{-13}
$\boldsymbol{k_3^0}$	4.3×10^{-3}
$\boldsymbol{k_4^0}$	1.1×10^{8}
$\boldsymbol{k_{-4}^0}$	3.1×10^{7}
$\boldsymbol{k_5^0}$	2.5×10^{-5}
$\boldsymbol{\alpha_2}$	0.63
$\boldsymbol{\alpha_3}$	0.10
$\boldsymbol{\alpha_5}$	0.73
$\boldsymbol{\alpha_6}$	0.10

Table S7. The evaluation parameters of the accuracy of the CO_2-to-formate kinetic model simulation.

Parameters	Value
Root Mean Squared Error (RMSE)	0.2872
Mean Absolute Error (MAE)	0.2477
Coefficient of Determination (R^2)	0.9893
Relative Error (RE%)	12.1607%
Pearson Correlation Coefficient (r)	0.9947

Table S8. Best-Fitted kinetic parameters of CO_2-to-formate.

Kinetic parameters	Value
$\boldsymbol{k_{B2}^0}$	1.64×10^{-11}
$\boldsymbol{k_{-B2}^0}$	2.43×10^{-9}
$\boldsymbol{k_{B3}^0}$	5.75×10^{-5}
$\boldsymbol{k_{-B3}^0}$	1.84×10^{-2}
$\boldsymbol{k_4^0}$	9.1×10^{11}
$\boldsymbol{k_{B5}^0}$	1.31×10^{-12}
$\boldsymbol{k_{-B5}^0}$	1.32×10^{-12}
$\boldsymbol{k_6^0}$	1.94×10^{-6}
$\boldsymbol{\alpha_1}$	0.3

α_2	0.1
α_4	0.5
α_5	0.1

Reference

1. X. Xin, Y. Li, L. Yu, W. Li, J. Li, R. Lu, Facile synthesis of Ag@C@Ag hybrid nanoparticles as SERS substrate, *Anal Bioanal Chem* 2021, 413, 5767–5777.
2. L. Huang, G. Gao, C. Yang, X.Y. Li, R.K. Miao, Y. Xue, K. Xie, P. Ou, C.T. Yavuz, Y. Han, G. Magnotti, D. Sinton, E.H. Sargent, X. Lu, Pressure dependence in aqueous-based electrochemical CO(2) reduction, *Nat Commun* 2023, 14, 2958.
3. J. Zhao, M. Jin, H. Huang, H. Guo, Y. Han, L. Huang, Y.-Y. Yuan, Q. Wang, J. Liang, X. Gao, J. Long, N. Tsubaki, X. Lu, Unlocking Atomic Degrees of Freedom in Liquid Metals for Accelerated Electrocatalytic Reactions, *ACS Catal*. 2025, 15, 3505–3514.
4. Y. Li, Y. Jiang, J. Dang, X. Deng, B. Liu, J. Ma, F. Yang, M. Ouyang, X. Shen, Application of distribution of relaxation times method in polymer electrolyte membrane water electrolyzer, *Chem. Eng. J.* 2023, 451, 138327.
5. Y. Lu, C.-Z. Zhao, J.-Q. Huang, Q. Zhang, The timescale identification decoupling complicated kinetic processes in lithium batteries, *Joule* 2022, 6, 1172−1198.
6. Y. Qu, K. Yang, W. Li, G. Wang, L. Xiao, G. Wang, L. Zhuang, Operando diagnosis of MEA-type CO_2 electrolyzer via distribution of relaxation times analysis, *ACS Energy Lett.* 2024, 9, 3042−3048.
7. X. Huo, G. Shan, L. Yang, L. Gao, Y. Wang, M. Zhang, Y. Fu, W. Li, J. Zhang, Impedance analysis of alkaline water electrolysis based on distribution of relaxation time, *Int J Hydrogen Energ* 2024, 53, 684–697.
8. H. Wu, B. Tian, W. Xu, K.K. Abdalla, Y. Kuang, J. Li, X. Sun, Pressure-Dependent CO2 Electroreduction to Methane over Asymmetric Cu–N2 Single-Atom Sites, *J. Am. Chem. Soc.* 2024, 146, 22266–22275.
9. S. Chinnathambi, M. Ramdin, T.J.H. Vlugt, Mass transport limitations in electrochemical conversion of CO_2 to formic acid at high pressure, *Electrochem* 3, 549−569 (2022).
10. M. Agliuzza, C.F. Pirri, A. Sacco, A comprehensive modeling for the CO_2 electroreduction to CO, *J Phys Energy* 2024, 6, 015004.
11. W. Deng, P. Zhang, B. Seger, J. Gong, Unraveling the rate-limiting step of two-electron transfer electrochemical reduction of carbon dioxide, *Nat Commun* 2022, 13, 803.
12. M. Dunwell, W. Luc, Y. Yan, F. Jiao, B. Xu, Understanding surface-mediated electrochemical reactions: CO_2 reduction and beyond, *ACS Catal* 2018, 8, 8121−8129.
13. S. Kumarampulakkil, A. Naikkath, R. Vinu, R. Srinivasan, Experimental studies and microkinetic analysis of electrochemical CO_2 reduction to CO on silver electrode, *Ionics* 2025, 31, 2277−2290.
